# Comparison of long-term outcomes of laparoscopic percutaneous internal ring suturing and classic open approach for inguinal hernia repair in children

**DOI:** 10.1308/rcsann.2024.0058

**Published:** 2024-07-25

**Authors:** S Kılıç

**Affiliations:** Private Gebze Yuzyil Hospital, Gebze, Kocaeli, Turkiye

**Keywords:** Children, Inguinal hernia, Laparoscopy, Minimally invasive surgery, Percutaneous internal ring suturing

## Abstract

**Introduction:**

Inguinal hernia repair is one of the most common surgeries in children. Recently, the use of minimally invasive techniques for inguinal hernia repair has increased in children. Laparoscopic percutaneous internal ring suturing (PIRS) is a technique described for paediatric inguinal hernia repair. The primary objective of this study is to demonstrate the efficacy and reliability of PIRS in paediatric patients through a comparative analysis with an open method.

**Methods:**

Between January 2017 and June 2023, records of patients who underwent surgery for inguinal hernia were retrospectively reviewed. In total 126 patients were included in the study. They were divided into two groups: 33 patients underwent PIRS (group 1) and 93 patients underwent open repair (group 2). Operation time, cost and complications were compared.

**Results:**

The mean age of the 126 patients was 3.23 ± 2.4 years. The mean unilateral operative time was 25.13 ± 5.32min in group 1 and 30.28 ± 4.73min in group 2, and there was a statistically significant difference in operative time (*p* < 0.001). Two patients in group 1 underwent surgery owing to patent processus vaginalis, whereas three patients in group 2 underwent surgery owing to metachronous hernia. There were no major complications such as bleeding requiring surgical intervention or conversion to an open approach during surgery in group 1. No recurrent hernia was observed in any of the 126 patients.

**Conclusions:**

PIRS offers a safe, easy-to-learn method with low complication rates. PIRS has a distinct advantage over open surgical repair because of its capacity to evaluate the contralateral inguinal ring.

## Introduction

Inguinal hernia is the protrusion of abdominal organs through the inguinal canal, resulting in swelling in the groin. The prevalence of inguinal hernia is 1–5%.^1^ However, this frequency increases significantly in connective tissue disorders, elevated intra-abdominal pressure and premature newborns.^2^ Paediatric inguinal hernia predominantly presents as an indirect type, unlike in adult patients. The pathophysiological explanation for the cause of hernia in children is the patency of the processus vaginalis, which is an embryological remnant.^3^

Inguinal hernia repair is the one of the most common operations in paediatric surgery practice. Classic surgical repair, involving the high ligation of the inguinal hernia sac, a technique dating back to the 18th century, has been successfully applied in paediatric patients for many years.^4^ However, in recent years, with the rapid development of minimally invasive techniques, different surgical approaches have been described and implemented in inguinal hernia repair. The initial classic three-port laparoscopic repairs in children reported high recurrence rates, prompting an exploration of alternative surgical techniques in paediatric patients.^5^ Laparoscopic percutaneous internal ring suturing (PIRS), described by Patkowski *et al* in 2006, draws attention in paediatric patients because of its rapidity, safety and very low recurrence rates.^6,7^

Although it is known that the recurrence rate in classic inguinal hernia repair is lower than in laparoscopic repair, it may be inadequate in detecting the presence of contralateral inguinal hernia.^8,9^ The development of inguinal hernia on the opposite side after unilateral repair can be disappointing for both the surgeon and the patient’s family. The main advantage of the PIRS method and other laparoscopic inguinal hernia repairs is the ability to visualise the contralateral inguinal canal during the same procedure to prevent the occurrence of metachronous inguinal hernias, which occur with a frequency ranging from 5% to 7.5%.^10,11^

This study aims to investigate the potential superiority of laparoscopic PIRS over traditional open repair for paediatric inguinal hernia repair. The hypothesis is that PIRS offers reduced recurrence rates, faster operation times and lower complication rates owing to its ability to visualise and address the contralateral inguinal canal. To test this hypothesis, we conducted a retrospective cohort study comparing patients who underwent PIRS and open repair, focusing on recurrence rates, complications, operation time and cost.

## Methods

### Study design

This study, following approval from the Ethics Committee of Istanbul Medipol University and the Institutional Review Board (Approval No. 2024/02/09), involved a retrospective review of data from 126 children who underwent inguinal hernia repair at Private Gebze Yuzyil hospital between January 2017 and July 2023. Consent for publication was obtained from the family of the patient. A retrospective review was conducted by examining the hospital information system, which identified a total of 156 patients who underwent surgery at the paediatric surgery clinic for inguinal hernia. The study excluded infants operated on within the newborn period and who were under 1 year of age, as well as those needing urgent surgery for incarcerated inguinal hernia. In addition, patients with specific chronic conditions linked to inguinal hernia formation, like Down syndrome, cystic fibrosis and cerebral palsy, were also excluded from the study. After excluding 30 patients, a total of 126 were included in the study. Of these patients, 33 were operated on using the PIRS method and were designated as group 1, whereas 93 underwent surgery using the conventional open surgical technique and were classified as group 2.

### Surgical procedures

#### Open approach

After the stages of general anaesthesia, draping and positioning, a transverse incision was made following the classic inguinal skin crease. After traversing the Camper and Scarpa's fasciae, the inguinal canal was gently dissected from the lateral sulcus. A small incision was made in the middle of the inguinal canal. Following suspension of the inguinal canal edges, the contents were delicately freed with a pick-up. Care was taken to identify and preserve the ilioinguinal nerve. In male children, the hernia sac was separated from the vas deferens and testicular vessel, followed by ‘high ligation’ at the proximal inguinal canal. In female patients, the hernia sac was opened to assess for any sliding organ. Repair of the sac was performed without high ligation if deemed necessary. In cases in which the internal ring was widened, a modified Ferguson reinforcement was applied. Closure was achieved sequentially for the canal and layers, concluding the procedure.

#### PIRS

All PIRS operations were performed by a single experienced surgeon. The method is described step-by-step below.

**Figure 1:**
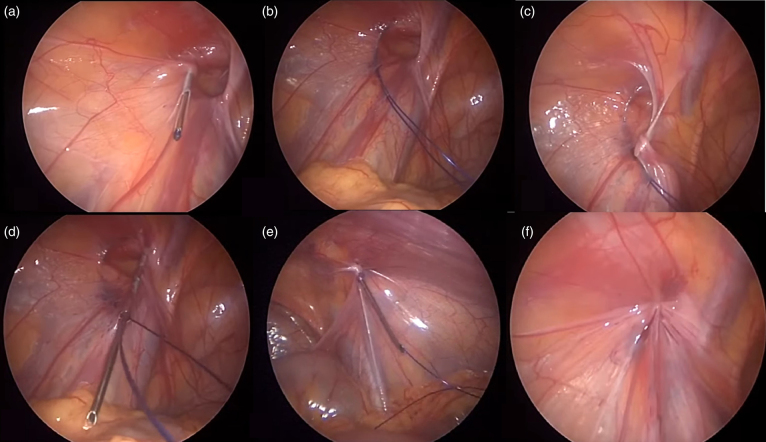
Steps in percutaneous internal ring suturing (PIRS). (a) Under laparoscopic visualisation, the injector needle is advanced obliquely through the skin overlying the projection of the inguinal canal. The peritoneum around the inguinal inner ring is gathered in a semi-lunar shape around the needle. (b) A non-absorbable suture (2-0 monofilament) is advanced through the needle. (c) The needle is wrapped around the remaining portion of the inguinal ring in a manner that will enclose the peritoneum for the second time. (d) The tip of the second inserted needle is passed through the space between the first prolene stitch. (e) A non-absorbable straight suture is advanced through the second needle and left in the peritoneum. (f) The first stitch is retrieved extracorporeally, completing a full loop with the second stitch. The thread is tied subcutaneously.

1.  **Entry of the trocar for laparoscopy (optics).** Entry with a Hasson (open) or Veress needle for the initial trocar was not preferred. Direct trocar entry technique was applied. This not only shortened the surgical time, but also prevented gas leakage. A 5-mm telescope was used for children up to 8 years old, whereas a 10-mm telescope was used for children older than 8 years or those who are relatively larger. A 30° angled telescope was selected for laparoscopic hernia repairs. The CO_2_ insufflation pressure in the peritoneal cavity was maintained between 8 and 12mmHg, adjusted according to the patient's age.
2.  **Intra-abdominal imaging.** Before starting hernia repair, all intra-abdominal organs were visualised. Both inguinal canals were visualised for metachronous hernias. The inguinal internal ring to be operated on was fixed to remain in the centre of the image.
3.  **Percutaneous passage of the needle threaded with suture into the inguinal canal.** Under laparoscopic visualisation, the passage of a needle threaded with suture into the internal inguinal ring is the most crucial surgical step. A slightly oblique-tipped injection needle (18 or 21 gauge) or a spinal needle may be preferred. We used a green injection needle (21 gauge) in children under 3 years of age and a pink injection needle (18 gauge) in children over 3 years old. The needle, threaded with a loop-shaped non-absorbable suture, is advanced under camera guidance from the edge of the internal inguinal ring towards the abdominal cavity. The peritoneum around the inguinal ring is gathered with this needle. Care should be taken not to injure the spermatic cord in males and the round ligament in females. After more than half of the peritoneum around the ring is gathered at the tip of the needle, the suture is advanced from the tip of the needle into the abdominal cavity.
4.  **Sending the second needle tip for the remaining half of the ring.** Using a needle of the same size again with a straight tip (not looped), a non-absorbable suture is pushed into the abdominal cavity from the opposite side of the internal inguinal ring under camera guidance. The remaining peritoneum of the internal ring is also gathered with this second needle. The tip of this second needle is pulled out from the point where the first suture entered the abdomen.
5.  **Retrieval of the first suture.** The second needle is passed through the loop of the first suture. The suture within the second needle is advanced, passing through the loop towards the abdominal cavity.
6.  **Pulling the first suture.** The first suture with a loop-shaped end is started to be pulled under the camera. Thus, the second suture follows the path of the first suture, forming a loop, and revolves around the internal inguinal ring by 360° (Figure 1).
7.  **Knotting stage.** After the first suture is pulled out of the abdomen, the second suture also exits through the loop. The first suture remains completely outside the abdomen. The second suture is prepared to be tied off with both ends outside the abdomen. Then, a knot is tied, and under camera visualisation, the closure of the internal ring is observed. The knot ends are cut short and hidden under the skin. This technique was applied as described by Patkowski.^6,7^

In six cases, an additional 5-mm or 100-mm port was placed in the lower abdominal quadrant to facilitate the use of additional instruments such as graspers or dissectors because of inadequate manoeuvrability with the needle. Anaesthesia and surgery times were meticulously recorded.

### Statistical analyses

Statistical analysis was conducted using IBM SPSS 22.0 statistical software (IBM Corporation, Chicago, IL, USA). Continuous variables were presented as mean ± sd and compared using the *t*-test. The normality of distribution was assessed using the Kolmogorov–Smirnov test. Student's *t*-test was used to compare normally distributed independent variables between groups, while the chi-squared test was employed for categorical data comparison. Statistical significance was set at *p* < 0.05.

## Results

The study included 126 patients: 33 in the study group and 93 in the control group. The mean age of the 126 patients was 3.23 (±2.41, range 1–11) years. Hernias were most commonly found on the right side (*n* = 72, 57%), followed by the left side (*n* = 46, 36%) and bilaterally (*n* = 8, 6%). There was a significant statistical difference between the ages of patients when compared between the two groups (*p* < 0.001). No significant statistical difference was observed when patients were compared based on sex and the side of hernia. Patient characteristics are detailed in Table 1. The mean operative time was 25.67 minutes in group 1 and 32.29 minutes in group 2, showing a statistically significant difference in operative time between the two groups. After excluding patients who underwent bilateral repair, a significant statistical difference was still found when comparing the operative times of unilateral hernia repairs between the two groups (*p* < 0.001).

**Table 1:** Patient characteristics

	Group 1 (PIRS) Mean ± sd	(Group 2) Open approach Mean ± sd	*p-*value
Number	33	93	
Age (years)	4.6 ± 2.1	2.7 ± 2.3	0.001*
Sex			0.328**
Male	20	65	
Female	13	28	
Side			0.644**
Right	19	53	
Left	13	33	
Bilateral	1	7	

*p* < 0.05 is significant.

*Student’s *t*-test.

**Chi-squared test.

PIRS = percutaneous internal ring suturing.

In group 1 (PIRS), there were no major complications such as bleeding requiring surgical intervention or conversion to open approach during surgery. All patients started with PIRS technique were able to complete repair using the same technique. There were no surgical wound infections in either group (Table 2).

**Table 2:** Operative and postoperative data

	Group 1 (PIRS) Mean ± sd	(Group 2) Open approach Mean ± sd	*p-*value
Cost ($)	80.7 ± 5.3 (9.4%)	42.3 ± 2.8	0.001*
Total operation time (minutes)	25.67 ± 6.09 (range 16–43)	32.29 ± 9.09 (range 21–70)	0.001*
Unilateral operation time (minutes)	25.13 ± 5.32 (range 16–40)	30.28 ± 4.73 (range 21–43)	0.001*
Recurrence	0	0	
Wound infection	0	0	
Operative bleeding	0	0	

*p* < 0.05 is significant.

*Student’s *t*-test.

PIRS = percutaneous internal ring suturing.

Owing to variations in the exchange rate in different years, the costs in the study were calculated in US dollars. The average surgical cost in group 1 was $80.70, whereas in group 2, it was $42.30, demonstrating a statistically significant difference between the two groups (Table 2).

All patients were discharged on the same day postoperatively (outpatient). There was no difference in hospital stay between the two groups.

Patients were called for a dressing change on the second postoperative day. Subsequently, follow-ups were conducted at 1 week, 1 month and 6 months. Scar formation and recurrence hernia were evaluated at the 6-month examination. No recurrence hernia was observed in either group. Among the patients who underwent open repair, 3 of 93 (3.22%) developed contralateral inguinal hernia and were repaired. Initial hernia development in these patients was on the left side in two cases and on the right side in one case. In addition, two patients (6%) in group 1 presented with synchronous open processus vaginalis and were operated on using the PIRS method.

## Discussion

Classic open repair is still commonly preferred for the repair of inguinal hernias in children. With advancements in minimally invasive surgery, inguinal hernia repair in children has begun to be performed using laparoscopic techniques.^4^ Discussions about whether laparoscopic hernia repair is safe for children have shifted towards studies showing its safety and feasibility. The focus is now on determining which method is preferred for hernia repair in children. When laparoscopic repair began to be performed on children, the same repair was performed on adult patients as well. This repair consisted of suturing around the internal inguinal ring with the use of three ports, known as the TAPP procedure.^12^ By contrast, several surgical techniques have been described for inguinal hernia repair in children, but many of them require suturing inside the abdomen and using multiple ports, which can be disadvantageous. It was noteworthy that the recurrence rate in laparoscopic repairs performed with three ports in children was high in the first series.^13^ Because the recurrence rate is higher in laparoscopy than in open repair, there have been authors who recommended dissecting the peritoneum around the inguinal canal during laparoscopic repair.^14,15^ Boo *et al* reported in their study that they reduced the recurrence rate by performing peritoneal dissection.^16^ After the classic repair using three ports, inguinal hernia repair was begun with the help of a single port. The PIRS method described by Patkowski and colleagues involves closing the internal ring inside the abdomen using only a laparoscopic-assisted suturing method. This method is simple, cost-effective and carries a low risk of injury to intra-abdominal organs.^5,6^

In their studies, Patkowski *et al* reported postoperative complications in 4 (2.9%) patients and recurrence in 3 (2.1%) patients, and Wolak and Patkowski reported similar results in their study, with complications in 2 (3.0%) children of 67 repairs performed in 55 children and recurrence in 1 (1.5%) child.^5,6^ In our study, none of the 33 patients who underwent PIRS were found to have recurrent hernia. Our results indicate a lower recurrence and complication rate compared with studies reporting hernia repair using the PIRS method by Patkowski and others.^17^ Similar to our study, Pogorelić *et al* recently reported that no recurrence was observed in 51 patients who underwent hernia repair using the PIRS method.^18^ In our study, we conducted regular patient follow-up and monitoring for a duration of 6 months. We believe this timeframe provides adequate observation time for detecting recurrent hernias. In addition, the institution where our study was conducted is not a training clinic. All surgeries were performed by a single surgeon with 15 years of experience.

The general consensus is that laparoscopic repairs conclude in a shorter time compared with open surgeries. The initial surgeries during the laparoscopic learning process tend to be longer. Matt *et al* presented a meta-analysis demonstrating that laparoscopy-assisted percutaneous repairs have shorter operation times than open surgery. In our study, we also found statistically significant shorter operation times with PIRS.^19^

The most common complication encountered with the PIRS method is bleeding caused by needle entry into the internal ring during external suturing. Bleeding is predominantly from the iliac vein. In our study, we did not observe any complications such as bleeding or associated hematoma. In a study conducted by Kang *et al*, among 43 patients who underwent PIRS repair, bleeding occurred during needle insertion in 5 cases (12%).^20^ Similarly, in a study by Kara *et al* involving 227 PIRS repairs, bleeding occurred in 6 cases (2.6%) following needle insertion.^21^ In our study, we did not encounter any bleeding related to needle insertion. These results are relatively low compared with the general literature. Furthermore, in another study presented by Zhang *et al*, complications such as hydrocele, scrotal oedema, groin pain and peritoneal rupture were reported.^22^ However, we did not experience these complications in our study. We attributed the low complication rate in our study to the limited number of patients compared with the literature.

One of the major concerns following unilateral inguinal hernia repair is the potential development of hernia on the contralateral side. The rate of contralateral inguinal hernia development has been reported to range between approximately 5.8% and 11.6% in various patient series.^23^ Although some traditional methods exist for detecting contralateral hernias during open surgeries, they are not commonly practised in routine clinical settings.^24^ Although the development of contralateral hernia has been associated with open patent processus vaginalis (PPV), Li *et al* argued in their study that not every open PPV resulted in hernia development.^25^ In our study, we repaired open internal inguinal rings laparoscopically. Particularly, there is a higher probability of contralateral inguinal hernia development after the occurrence of inguinal hernia on the left side. In our study, three patients (6%) developed contralateral inguinal hernia following open surgery. Two of these patients had previously undergone surgery for left inguinal hernia. These findings are consistent with the literature. In addition, in two patients who underwent repair with PIRS during the same procedure, open contralateral inguinal hernia was detected and repaired.

### Study limitations

Several limitations are present in this study. First, although the number of patients included is sufficient for evaluation, patient selection depends heavily on age. Given that the PIRS method is a laparoscopic procedure, our study focused on patients aged three and above. Exclusion of neonatal and incarcerated inguinal hernia cases aimed to enhance group homogeneity. It is also recognised that laparoscopic surgeries entail a learning curve. In our study, there was a time disparity between the first and last patients, yet subgroup analysis was not pursued because of the limited number of patients. The average follow-up period for patients operated with the PIRS method in this study is shorter than those operated using the open method, which is one of the limitations of this study.

## Conclusion

In this study, the outcomes of paediatric inguinal hernia repair using PIRS were compared with the classic open repair. Although the classic approach is still commonly preferred, PIRS has gained popularity in recent years as a simple laparoscopic and percutaneous method. One of the main advantages of PIRS is its ability to control the contralateral internal ring and provide a faster repair. After longer-term series and meta-analyses, the PIRS method may be the primary choice for surgical repair in children.

## Data availability

The data sets generated and analysed during the current study are not publicly available owing to patients’ privacy but are available from the corresponding author on reasonable request.

## Ethical approval and patient consent

This study protocol was approved by İstanbul Medipol University, Faculty of Medicine, Non-Interventional Clinical Research Ethics Committee (Date: February 20, 2024, No: E-10840098-202.3.02-1437). The study was performed in accordance with the ethical standards laid down in the 1964 Declaration of Helsinki and its later amendments. Consent for publication was obtained from the patient’s family.
